# Exogenous C-type natriuretic peptide restores normal growth and prevents early growth plate closure in its deficient rats

**DOI:** 10.1371/journal.pone.0204172

**Published:** 2018-09-20

**Authors:** Keisho Hirota, Mayumi Furuya, Naomi Morozumi, Kazunori Yoshikiyo, Takafumi Yotsumoto, Toshimasa Jindo, Ryuichi Nakamura, Koichiro Murakami, Yohei Ueda, Takeshi Hanada, Hiroko Sade, Sayaka Yoshida, Kei Enomoto, Yugo Kanai, Ichiro Yamauchi, Takafumi Yamashita, Yoriko Ueda-Sakane, Toshihito Fujii, Akihiro Yasoda, Nobuya Inagaki

**Affiliations:** 1 Department of Diabetes, Endocrinology and Nutrition, Kyoto University Graduate School of Medicine, Kyoto, Japan; 2 Asubio Pharma Co. Ltd. Kobe, Japan; 3 Medical Innovation Center, Kyoto University Graduate School of Medicine, Kyoto, Japan; Universite de Nantes, FRANCE

## Abstract

Signaling by C-type natriuretic peptide (CNP) and its receptor, natriuretic peptide receptor-B, is a pivotal stimulator of endochondral bone growth. We recently developed CNP knockout (KO) rats that exhibit impaired skeletal growth with early growth plate closure. In the current study, we further characterized the phenotype and growth plate morphology in CNP-KO rats, and the effects of exogenous CNP in rats. We used CNP-53, an endogenous form of CNP consisting of 53 amino acids, and administered it for four weeks by continuous subcutaneous infusion at 0.15 or 0.5 mg/kg/day to four-week old CNP-KO and littermate wild type (WT) rats. We demonstrated that CNP-KO rats were useful as a reproducible animal model for skeletal dysplasia, due to their impairment in endochondral bone growth. There was no significant difference in plasma bone-turnover markers between the CNP-KO and WT rats. At eight weeks of age, growth plate closure was observed in the distal end of the tibia and the calcaneus of CNP-KO rats. Continuous subcutaneous infusion of CNP-53 significantly, and in a dose-dependent manner, stimulated skeletal growth in CNP-KO and WT rats, with CNP-KO rats being more sensitive to the treatment. CNP-53 also normalized the length of long bones and the growth plate thickness, and prevented growth plate closure in the CNP-KO rats. Using organ culture experiment of fetal rat tibia, gene set enrichment analysis indicated that CNP might have a negative influence on mitogen activated protein kinase signaling cascades in chondrocyte. Our results indicated that CNP-KO rats might be a valuable animal model for investigating growth plate physiology and the mechanism of growth plate closure, and that CNP-53, or its analog, may have the potential to promote growth and to prevent early growth plate closure in the short stature.

## Introduction

C-type natriuretic peptide (CNP) is a member of the natriuretic peptide family, and a selective ligand for natriuretic peptide receptor-B (NPR-B) [1]. We and another group have reported that CNP and NPR-B knockout (KO) mice exhibit growth failure due to impaired endochondral bone growth [2–4]. In contrast, transgenic (Tg) mice with systemic or cartilage-specific overexpression of CNP show a skeletal overgrowth phenotype [5–8]. In humans, loss-of-function mutations in the NPR-B gene [9, 10], as well as the CNP gene [11], are reported to cause an autosomal dominant short stature. Conversely, gain-of-function mutations in the gene encoding NPR-B, and increased CNP expression by balanced chromosomal translocations reportedly cause skeletal-overgrowth [12, 13]. These findings indicate that CNP/NPR-B signaling is a pivotal stimulator of endochondral bone growth in humans, as well as in rodents.

We recently developed CNP-KO rats using zinc-finger nuclease technology [14]. The CNP-KO rats exhibit impaired skeletal growth, caused by a deficiency in endochondral ossification, which agrees with the findings in CNP-KO mice. However, in contrast to the early death in CNP-KO mice, CNP-KO rats survive beyond one year, and remain shorter than wild-type (WT) rats throughout their life. Furthermore, because rats have a larger body size and longer growth period than mice [15], the CNP-KO rats may be suitable for long-term morphological analyses of skeletal tissues, making them a potential animal model for short stature.

We have previously reported that overexpression of CNP in the cartilage or liver almost completely rescues skeletal growth failure in a mouse model for achondroplasia [5, 7]. However, these transgenes are expressed prior to parturition, or just after birth, and we have only been able to demonstrate a preventive effect of CNP on skeletal growth. Exogenous administration of CNP-22, the 22-amino acid form of CNP, also stimulates skeletal growth in both normal mice and the achondroplasia mouse model, but requires a high-dose intravenous infusion (1.44 mg/kg/day) because of its short plasma half-life [7].

To further evaluate the skeletal features of CNP-KO rats, and to investigate the therapeutic effect of CNP on skeletal growth, we administered CNP to CNP-KO and WT rats. We used CNP-53, the 53-amino acid form, since this molecular form is predominant compared with CNP-22 in both central and peripheral tissues [16] and has a higher resistance to neutral endopeptidase (NEP) than CNP-22 [17]. In the current report, we show a dose-dependent effect of CNP-53 on skeletal growth, and its effects on growth plate thickness and closure in CNP-KO and WT rats. Organ culture experiments were also carried out using tibiae from fetal rats, and DNA microarray and gene set enrichment analyses were conducted to identify the signaling pathways of CNP.

## Materials and methods

### Animals

All animal care and experiments were conducted under the guidelines for animal experiments of Kyoto University and Asubio Pharma Co., Ltd., and approved by the Animal Research Committee of Kyoto University (Med Kyo 17218), and by the committee for ethics in animal experiments of Asubio Pharma Co., Ltd. CNP-KO rats were generated as previously reported [14]. The CNP-KO rats had a homozygous Δ11 deletion of nucleotides 192–202 (NM_053750.1), which generated a frame shift, and a premature stop codon at nucleotides 275–277 (NM_053750.1) in the natriuretic peptide precursor C (*Nppc*) gene. Male and female CNP-KO rats and littermate WT rats were used; in some experiments, a mismatch of sample numbers between the two genotypes existed. For RT-PCR analysis, we used three-week-old male CNP-KO rats with homozygous Δ774 mutation, which caused massive deletion within the *Nppc* gene that included the translation initiation site. We previously confirmed that both Δ11 and Δ774 homozygous lines had CNP deficiency and exhibited almost identical impaired skeletal phenotype [14]. Genotyping of CNP-KO rats were performed by PCR analysis using genomic DNA from tail biopsy, as described in our previous report [14]. The animals were housed in a humidity and temperature-controlled environment with an automatic 12-hour light/dark cycle. They were fed a standard pelleted lab chow diet (CRF-1; Oriental Yeast Co., Ltd., Japan) and tap water *ad libitum*.

### Peptide

Human CNP consisting of 53 amino acids (CNP-53) with two amino acid substitutions in the N-terminal region compared with the rat CNP-53, but with similar biological activity in rats [18], was used in the current studies. The human CNP-53 was produced in *Escherichia coli* using recombinant DNA methodologies, purified by high-performance liquid chromatography, and its amino acid composition was verified by electrospray ionization mass spectrometry. The purity of the CNP-53 was greater than 95%.

### Quantitative RT-PCR

Total RNA was extracted from the tibial growth plate of three-week-old CNP-KO and WT rats using Isogen reagent (Nippon Gene, Tokyo, Japan). Quantitative RT-PCR was subsequently performed using a TaqMan^®^ RT-PCR assay with primers specific for *Nppc*, *Npr2* (the gene encoding NPR-B) and *Npr3* (the gene encoding NPR-C, the clearance receptor for natriuretic peptide [1]), which were obtained from the manufacturer (Applied Biosystems). We also conducted RT-PCR for osteocrin, an endogenous ligand for NPR-C [19], using SYBR Green qPCR with following primers: rat osteocrin forward: CCGAGTTTGGAGCAGAAAGC; reverse: CAAGCTCCGTGGCTGACTTC, and rat beta actin forward: AGGCCAACCGTGAAAAGATG; reverse: CACAGCCTGGATGGCTACGT.

### CNP-53 treatment of CNP-KO and WT rats

Four-week-old male and female CNP-KO rats and WT rats received a constant subcutaneous (sc) infusion of CNP-53 of approximately 0.15 or 0.5 mg/kg/day, or vehicle, for three or four weeks using an osmotic mini-pump (ALZET^®^ osmotic pump 1002, Durect Corporation, CA, USA). Since continuous expression in the liver of CNP-Tg mice results in elevated levels of circulating CNP, which in turn promotes skeletal growth [6], we chose to mimic this condition in the current study by administering the CNP-53 by constant sc infusion. We recently reported that repeated sc administration of a CNP analog at 0.1–0.5 mg/kg/day for approximately four weeks significantly stimulates skeletal growth in normal mice [20]. Based on these findings, we selected 0.5 mg/kg/day as a high dose of CNP-53, and 0.15 mg/kg/day as a low dose, being approximately one-third the amount of the high dose. The CNP-53 was dissolved in 0.03 M acetate buffer (pH 4.0), 1% benzyl alcohol, and 10% purified sucrose for use as dosing solutions. The concentrations of the dosing solutions were based on body weight data of CNP-KO and WT rats in our previous study [14], and calculated using the average body weight of CNP-KO and WT rats at four to six weeks of age, and six to eight weeks of age. The osmotic mini-pumps using the dosing solutions for four to six weeks of age were implanted in the rats at the start of the experiments. The pumps were replaced after 14 days of infusion with new ones using the dosing solutions for rats six to eight weeks of age.

### Body weight and body length

Body weight and naso-anal body length of the CNP-KO and WT rats were evaluated weekly during the treatment period. The naso-anal length was measured using a ruler while the rats were under anesthesia of an intraperitoneal (ip) injection of 30 mg/kg pentobarbital (Sigma-Aldrich Japan, Tokyo).

At the end of the 4-week infusion treatment, the female CNP-KO and WT rats that had received either vehicle or 0.5 mg/kg/day of CNP-53 were deeply anesthetized with an ip injection of pentobarbital, and blood was collected from the vena cava, followed by the harvesting of the femur, tibia, calcaneus, and vertebra for subsequent analyses.

### Skeletal analysis and histology

Skeletal analysis was performed as previously described [7]. Briefly, rats were subjected to soft x-ray analysis at 30 kVp, 5 mA for 1 min using a Softron Type SRO-M5 (Softron, Tokyo, Japan), and the lengths of the bones were measured from the images on the x-ray film.

The femur, tibia, and calcaneus were dissected from the rats, fixed in 10% neutral buffered formalin, and decalcified in mixture of 10% formic acid and formalin solution for a week at room temperature. The samples were embedded in paraffin, sectioned, and stained with hematoxylin and eosin (HE) and further with or without alcian blue. The thickness of the growth plates at the proximal and distal ends of the femur and tibia, and at the calcaneus were measured under light microscopy. The thickness of the growth plate was measured at 300 μm intervals for nine sites on the proximal end of femur, and for five sites on the distal end of tibia and calcaneus. The mean thickness of the measured sites was considered as the growth plate thickness for each individual. For the distal end of femur and the proximal end of tibia, the thickness of the growth plate at the mid site was measured and analyzed.

The thickness of the hypertrophic and non-hypertrophic zones of the growth plate at the proximal end of tibia was also measured. Using the HE stained specimens, each zone in the growth plate was distinguished grossly based on the morphology of the chondrocytes constituting the respective zones. The resting, proliferative, and hypertrophic zones were located continuously from the epiphyseal to diaphyseal regions of the growth plate. The resting zone was defined as the area containing irregularly arranged round cells, and the proliferative zone was determined as the area containing flat-shaped and columnar-arranged chondrocytes. The hypertrophic zone was defined by the area that contained round chondrocytes with enlarged cytoplasm. We considered the non-hypertrophic zone to be the combination of the resting and proliferative zones. The delineation between the proliferative and hypertrophic zones was performed by two independent observers and was confirmed to have general reproducibility, and the thickness of the hypertrophic and non-hypertrophic zones was measured at one representative site in each specimen.

### Bone microstructural analysis

Microstructural analysis of the distal metaphysis of the femurs was performed by micro computed tomography (micro-CT) using an SMX-100CTSV3 (Shimadzu Co., Kyoto, Japan) as previously described [21]. A 2.0 mm region located 0.5 mm proximal of the distal growth plate of femur was set as the scan area. The findings from the micro-CT scans were used to determine the bone volume fraction (BV/TV), trabecular thickness (Tb.Th), and trabecular number (Tb.N). These structural indices were calculated using the three-dimensional trabecular bone analysis software TRI/3D-BON (Ratoc System Engineering Co., Tokyo, Japan).

### Plasma analysis

All blood samples were treated with 1/100 volume of 10% EDTA. After centrifugation at 12,000 rpm for 5 minutes at 4°C, the plasma samples were collected, and the amount of CNP-53 was quantified by radioimmunoassay (RIA). The CNP RIA was conducted using antiserum that recognized the 17-member, internal disulfide ring of CNP, with [^125^I]-labeled [Tyr^0^]-CNP(1–22) as a tracer [20]. Human CNP-53 was used as a standard control in the assay. The remaining portion of the plasma samples were subjected to estimation of bone metabolic markers. The markers were measured as follows: osteocalcin, using an Osteocalcin, Rat, Enzyme Immunoassay Kit, BT-490 (Biomedical Technologies, Inc., MA, USA), and osteoclast-derived-tartrate-resistant acid phosphatase 5b (TRAP5b), using a RatTRAP™ (TRAcP 5B) ELISA kit (SB-TR102, Immunodiagnostic Systems, UK).

### Organ culture and RNA isolation

Rats with heterozygous CNP-KO allele were mated to obtain fetal WT and homozygous CNP-KO rats. At embryonic day 18.5 (E18.5), tibiae were aseptically dissected from fetal rats, then static culture was performed in BGJb medium (Thermo Fisher Scientific, MA, USA) containing 6 mg/mL BSA (Wako Pure Chemical Industries, Osaka, Japan), 150 μg/mL ascorbic acid (Wako Pure Chemical Industries), and 100 units/mL penicillin and 100 μg/mL streptomycin (Wako Pure Chemical Industries). No serum was added to the medium. Genotyping was performed using a forearm of fetal rats within 24 hours from the start of the culture. After a pre-incubation for 24 hours, tibial explants from WT and homozygous CNP-KO rats were divided into DMSO and CNP (10^−7^ M) treated groups. Firstly, whole lengths of tibiae were measured at the start and after a 48-hour incubation period using a linear ocular scale mounted on a dissecting microscope. Next, to isolate RNA, tibial explants from WT and homozygous CNP-KO rats were incubated for 24 hours with DMSO or CNP (10^−7^ M), and then, hypertrophic zones of proximal growth plates of tibiae were dissected out using a dissecting microscope as reported previously [22]. Following, total RNA was isolated from the hypertrophic zones using RNeasy Micro Kit (Qiagen, Hilden, Germany), according to manufacturer’s protocol.

We have previously reported that CNP inhibits mitogen-activated protein kinase/extracellular signal-regulated kinase (MAPK/ERK) signaling activated by fibroblast growth factor (FGF) [5]. In order to estimate the involvement of this pathway, we investigated the effect of MAPK/ERK kinase 1/2 (MEK 1/2) inhibitor, U0126 (Calbiochem^®^, Merck KGaA, Darmstadt, Germany). Organ culture of tibiae from fetal WT rats (E18.5) was performed with DMSO or U0126 (20 μM), and the estimation of the growth of tibial explants and the extraction of total RNA from hypertrophic zones were performed as described above.

### DNA microarray analysis

The total RNA extracted from hypertrophic zones of tibial explants from fetal rats was then amplified and labelled with cyanine 3 using the one-color Low Input Quick Amp Labeling Kit (Agilent Technologies, CA, USA) according to the manufacturer’s instructions. The labelled cRNA was fragmented and hybridized to the Agilent SurePrint G3 Rat GE 8x60K Microarray or Agilent SurePrint G3 Rat GE 8x60K Ver. 2 Microarray. After the microarrays were washed, they were scanned with an Agilent DNA Microarray Scanner. The intensity value for each scanned feature was quantified with the Agilent Feature Extraction software, which subtracted the background. Agilent GeneSpring GX version14.9 was used for normalization as follows. First, signal intensities less than 1.0 were set to 1.0. Each chip was then normalized to the 75th percentile of the measurements taken from that chip. Finally, the normalized intensities were log2-transformed. The microarray data have been submitted to the National Center for Biotechnology Information Gene Expression Omnibus (GEO) and are available under the accession number GSE112639.

To determine whether gene sets of interest were statistically enriched among up- and down-regulated genes, we analyzed our non-redundant list of genes using GSEA 3.0 for pre-ranked lists [23].

### Statistical analysis

Data were expressed as means ± SE. Statistical analysis was performed using either Student’s *t*-test or Dunnett’s multiple comparison test. *P* values less than 0.05 were considered statistically significant.

## Results

### CNP-53 rescued growth of CNP-KO rats

We administered CNP-53 at 0.5 mg/kg/day to both male and female CNP-KO rats for three weeks. As shown in Table 1, the administration of CNP-53 similarly increased the body lengths and body weights in both the male and female CNP-KO rats. Based on this finding, we used female CNP-KO and WT rats in the subsequent studies.

**Table 1:** Effect of CNP-53 on body length and body weight in male and female CNP-KO rats.

Sex	CNP-53 Dose (mg/kg/day)	n	Body Length (cm)						Body Weight (g)					
			Day 1			Day 22			Day 1			Day 22		
Male	0 (Vehicle)	6	10.6	±	0.2	14.3	±	0.3	34.7	±	2.5	91.5	±	6.9
						(136	±	4%)				(269	±	24%)
	0.5	7	10.4	±	0.4	18.4	±	0.5	31.6	±	3.6	113.2	±	7.5
						(178	±	4%)				(375	±	28%)
Female	0 (Vehicle)	5	10.8	±	0.2	14.0	±	0.1	38.8	±	2.3	84.4	±	2.8
						(130	±	2%)				(219	±	6%)
	0.5	6	10.3	±	0.2	17.9	±	0.3	30.8	±	2.8	102.2	±	3.3
						(173	±	3%)				(342	±	26%)

Each value represents the mean ± SE. The values in the parentheses are the percent changes relative to Day 1. Day 1 and Day 22 show the first and 22nd day the drug was administered, respectively.

**Fig 1:**
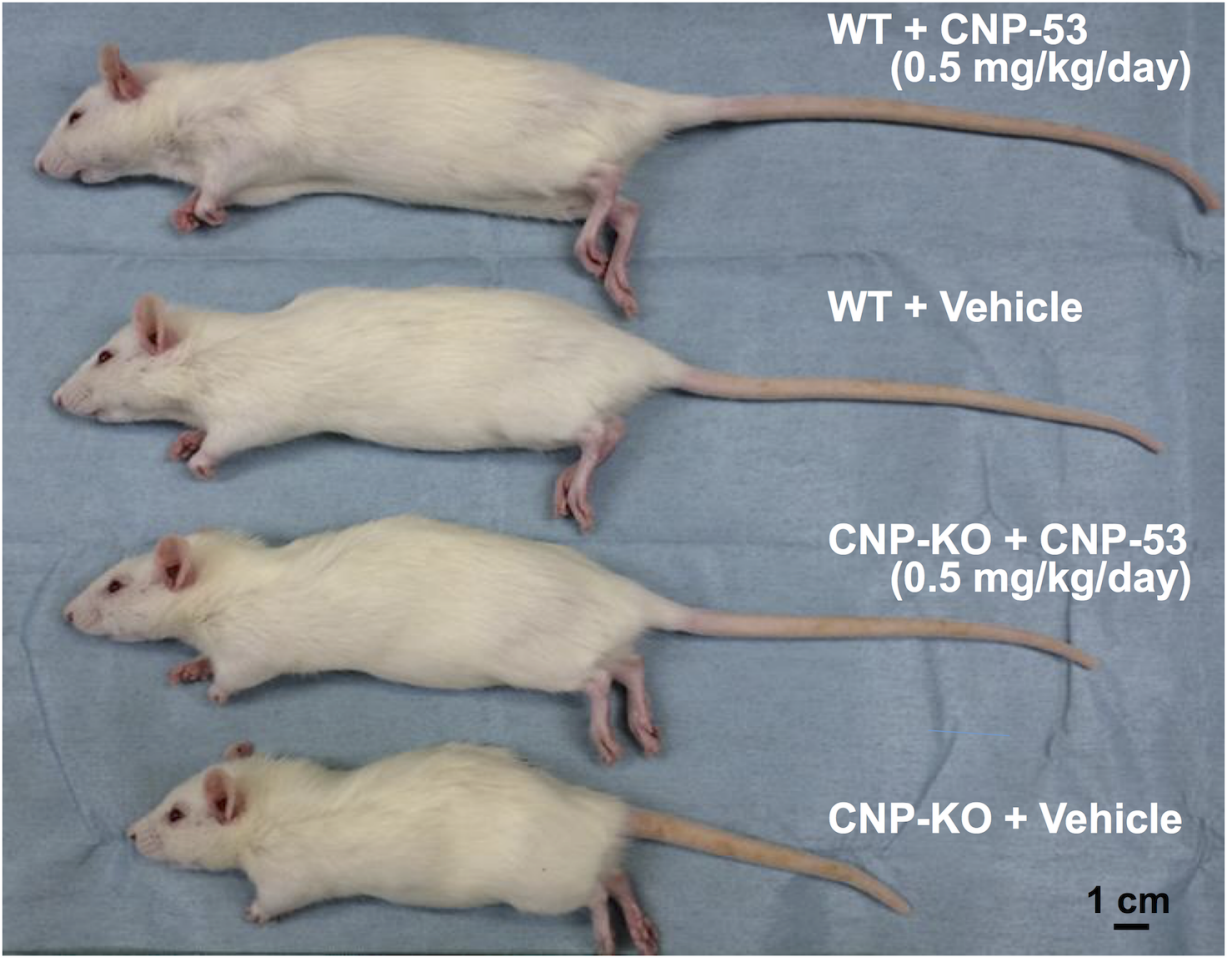
Gross appearance of 8-week-old female WT and CNP-KO rats that received vehicle or CNP-53 (0.5 mg/kg/day) for 28 days. The scale bar indicates 1.0 cm.

Female CNP-KO and WT rats received approximately 0.15 or 0.5 mg/kg/day of CNP-53 for four weeks. Fig 1 shows the gross appearance of CNP-KO and WT rats that received CNP-53 (0.5 mg/kg/day) or vehicle. The small phenotype of CNP-KO rats was nearly completely rescued by CNP-53 treatment. The WT rats that received CNP-53 were also larger than those that received the vehicle.

**Fig 2:**
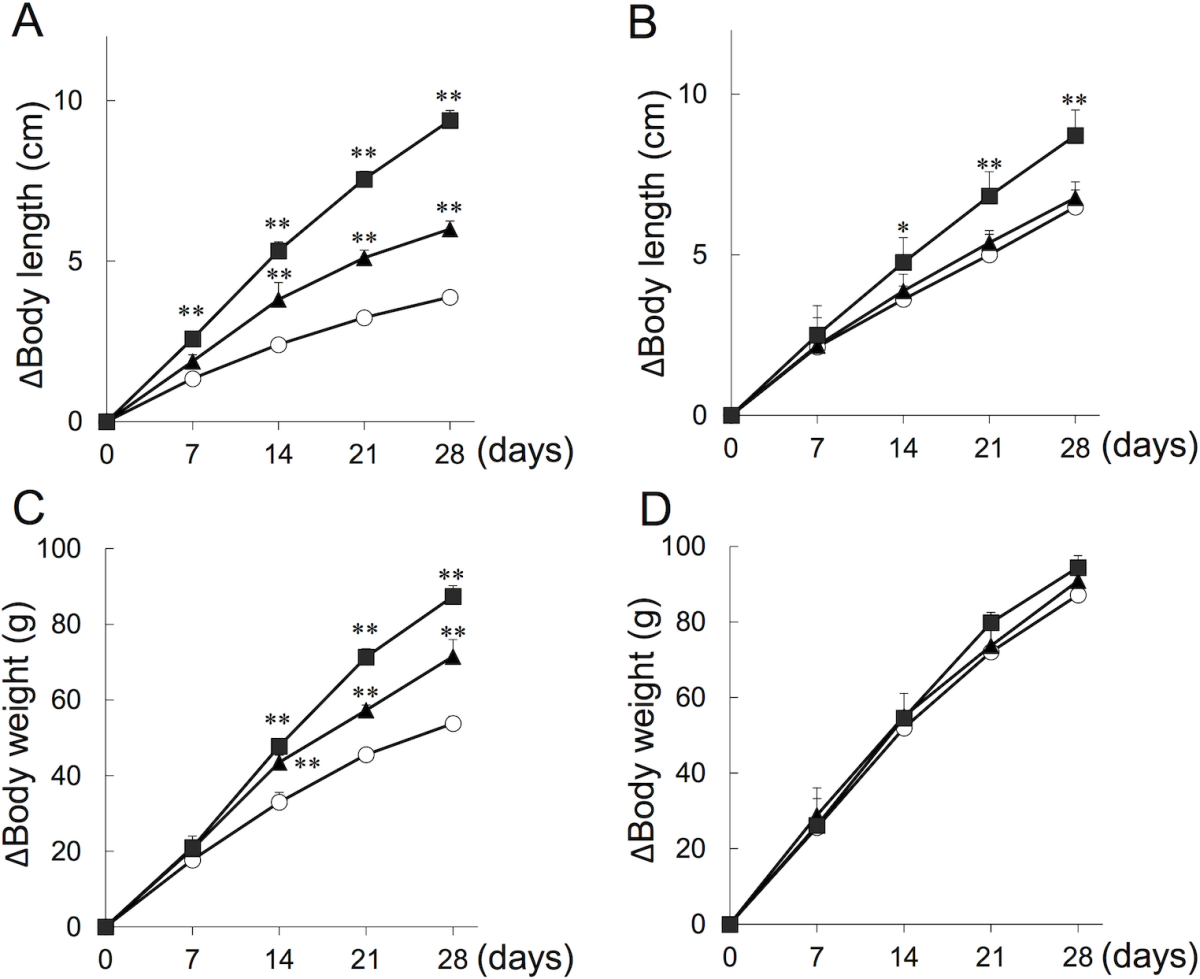
Changes in body length and body weight of female WT and CNP-KO rats that received vehicle or CNP-53 for 28 days. Changes in naso-anal body length (Δ body length) in CNP-KO rats (A) and WT rats (B), and changes in body weight (Δ body weight) in CNP-KO rats (C) and WT rats (D). Each value shown is the mean + SE. Open circles, vehicle (n = 5 and 7 in CNP-KO and WT rats, respectively); closed triangles, CNP-53 0.15 mg/kg/day (n = 4 in the both genotypes); closed squares, CNP-53 0.5 mg/kg/day (n = 6 in the both genotypes). *, *p* < 0.05 vs. vehicle-treated group, **, *p* < 0.01 vs. vehicle-treated group based on Dunnett’s multiple comparison test.

The changes in body length and body weight during the treatment period for female CNP-KO and WT rats are shown in Fig 2 and Table 2. The body length and body weight of CNP-KO rats were significantly less than those of WT rats, consistent with our previous report [14]. The CNP-KO rats were longer when treated with CNP-53, and the extent of the response was dose dependent. CNP-53 treatment also increased the body length of WT rats, but a statistically significant effect was observed only for those that received 0.5 mg/kg/day of CNP-53.

**Table 2:** Effect of CNP-53 on body length and body weight in female CNP-KO and WT rats.

Genotype	CNP-53 (mg/kg/day)	n	Body Length (cm)					
			Day 1		Day 29		Increment	
CNP-KO	0	5	10.8	± 0.2 ^##^	14.6	± 0.1 ^##^	3.9	± 0.2 ^##^
	0.15	4	10.5	± 0.1	16.5	± 0.2 **	6.0	± 0.1**
	0.5	6	10.3	± 0.2	19.7	± 0.3 **	9.4	± 0.3**
WT	0	7	13.3	± 0.3	19.8	± 0.1	6.5	± 0.3
	0.15	4	13.5	± 0.2	20.3	± 0.1	6.8	± 0.1
	0.5	6	13.1	± 0.5	21.9	± 0.2**	8.7	± 0.5**
Genotype	CNP-53 (mg/kg/day)	n	Body Weight (g)					
			Day 1		Day 29		Increment	
CNP-KO	0	5	38.8	± 2.3 ^##^	92.6	± 3.0 ^##^	53.8	± 0.9 ^##^
	0.15	4	40.5	± 0.8	112.0	± 1.9**	71.5	± 2.2**
	0.5	6	30.8	± 2.8	118.2	± 3.4**	87.4	± 2.8**
WT	0	7	49.9	± 2.6	137.1	± 2.1	87.1	± 3.2
	0.15	4	50.5	± 0.9	141.4	± 1.4	90.9	± 1.4
	0.5	6	46.5	± 4.6	141.0	± 3.0	94.4	± 3.1

The values are the means ± SE. Day 1, The initial day of administration of the CNP-53 or vehicle; Day 29, The final day of the drug administration

^##^, *p* < 0.01 vs. WT rats based on Student’s *t*-test.

**, *p* < 0.01 vs. vehicle (0 mg/kg/day) group based on Dunnett’s multiple comparison test.

In CNP-KO rats, the increase in body length with treatment of CNP-53 at 0.15 mg/kg/day was significantly larger than that observed for the group of CNP-KO rats receiving vehicle only (6.0 ± 0.1 cm vs. 3.9 ± 0.2 cm, respectively), and the increase was similar to that of the vehicle-treated WT rats (6.5 ± 0.3 cm). Treatment with the high dose of CNP-53 resulted in greater increased body length gains, with the average final body length in the group being 19.7 ± 0.3 cm, which was almost identical to that of vehicle-treated WT rats being 19.8 ± 0.1 cm (Table 2).

CNP-53 also significantly increased the body weight gain in the CNP-KO rats, but did not have a similar result in the WT rats. The increase in total body weight gain for the CNP-KO rats treated with 0.5 mg/kg/day of CNP-53 was as large as that seen in the vehicle-treated WT rats; however, the final body weight was still significantly smaller (Table 2).

Since the dosing of CNP-53 was based on our previous body weight data for CNP-KO rats, we confirmed the actual doses of CNP-53 administered to the female rats according to weekly body weight measurements. In the groups that were intended to receive 0.15 mg/kg/day of CNP-53, the average doses during the four-week treatment period were 0.14 mg/kg/day in the CNP-KO rats, and 0.15 mg/kg/day in WT rats. In the groups targeted to receive 0.5 mg/kg/day of CNP-53, the average doses were 0.52 mg/kg/day and 0.54 mg/kg/day in CNP-KO and WT rats, respectively, indicating that the dosing rates of CNP-53 were appropriately set. The plasma CNP concentrations at steady state in the CNP-KO and WT rats treated with the high dose of CNP-53 were 1.12 ± 0.94 and 1.37 ± 0.78 ng/mL, respectively (n = 4 and 5 in CNP KO and WT rats, respectively, and each value is shown in S1 Table).

### Effect of CNP-53 on bone length

**Fig 3:**
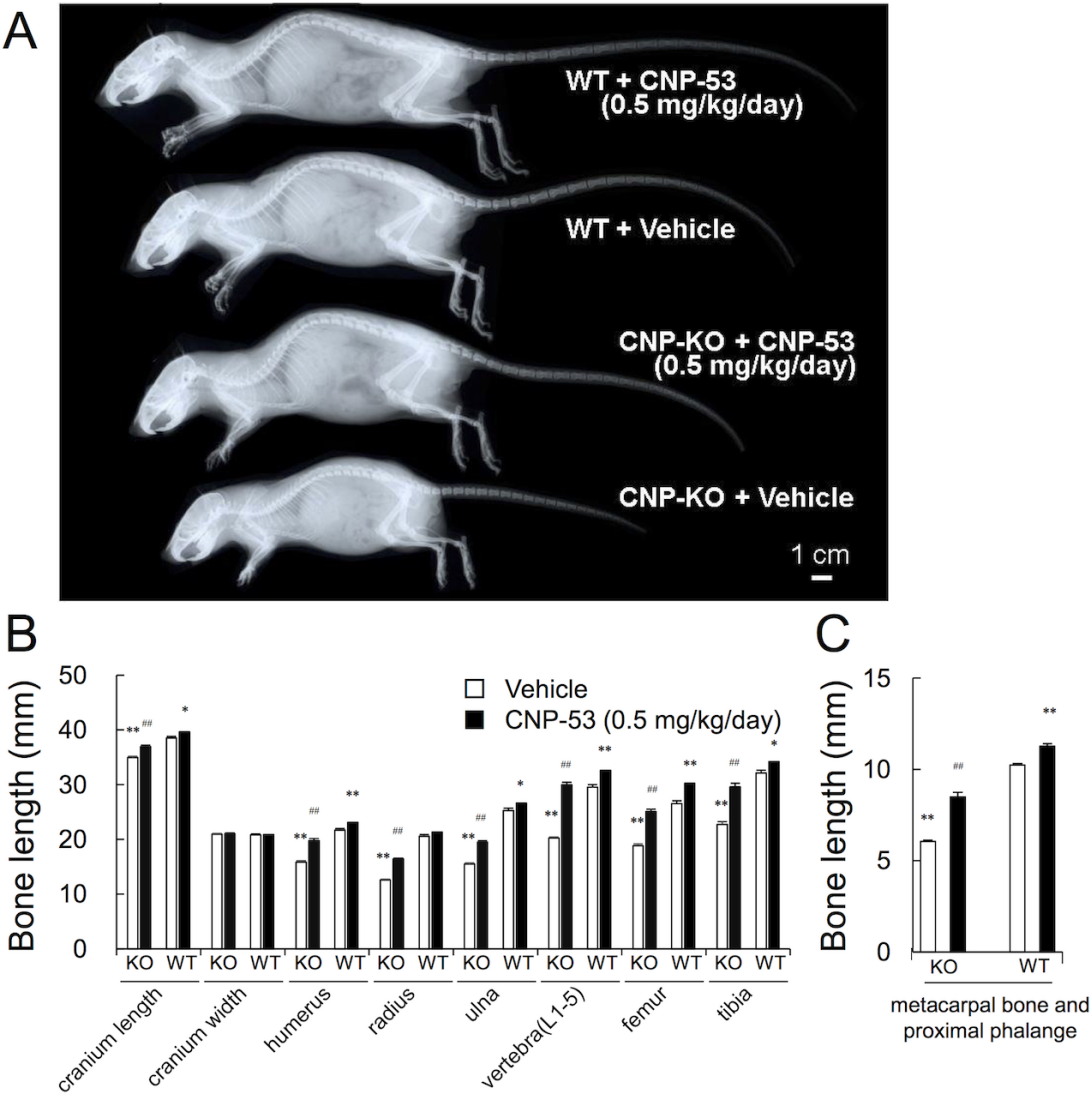
Skeletal phenotypes of CNP-KO and WT rats and the effect of CNP-53 on bone length. (A) Soft x-ray images of 8-week-old CNP-KO and WT rats. (B), (C) Bone lengths (B) and especially those in the paw (C) of rats at the age of 8 weeks as measured from soft x-ray films. The bars indicate the mean + SE. Open bars, vehicle (n = 5 in the both genotypes); closed bars, CNP-53 (0.5 mg/kg/day, n = 6 in the both genotypes). KO, CNP-KO rats; WT, WT rats. *, *p* < 0.05 vs. WT rats that received vehicle group. **, *p* < 0.01 vs. WT rats that received vehicle group. ^##^, *p* < 0.01 vs. KO rats that received vehicle group, based on the Student’s *t*-test.

Skeletal phenotypes of CNP-KO and WT rats were analyzed from soft X-ray pictures after four weeks of drug administration. Female rats received either vehicle or a high dose of CNP-53 were subjected to analysis. The CNP-KO rats were shorter than the WT rats due to impaired skeletal growth (Fig 3A). In the CNP-KO rats, each bone formed through endochondral ossification was significantly shorter than in the WT rats, consistent with findings previously reported [14]. The widths of the crania in CNP-KO rats were almost identical to those observed in WT rats. In contrast, the lengths of the crania, humeri, radii, ulnae, vertebrae, femurs, and tibiae in CNP-KO rats were significantly shorter compared to those in WT rats, and CNP-53 treatment significantly increased all these lengths (Fig 3B). CNP-53 treatment also elongated all the above bones of the WT rats except radii (Fig 3B). As the humans with heterozygous CNP mutations reportedly had brachydactyly [11], we also investigated the lengths of bones in paw. As depicted in Fig 3C, the sum length of proximal phalange and metacarpal bone in CNP-KO rats was significantly smaller than that in WT rats. CNP-53 treatment increased the sum length of proximal phalange and metacarpal bone in CNP-KO rats and also increased it in WT rats as well (Fig 3C).

### Growth plate thickness and histology after CNP-53 treatment

**Fig 4:**
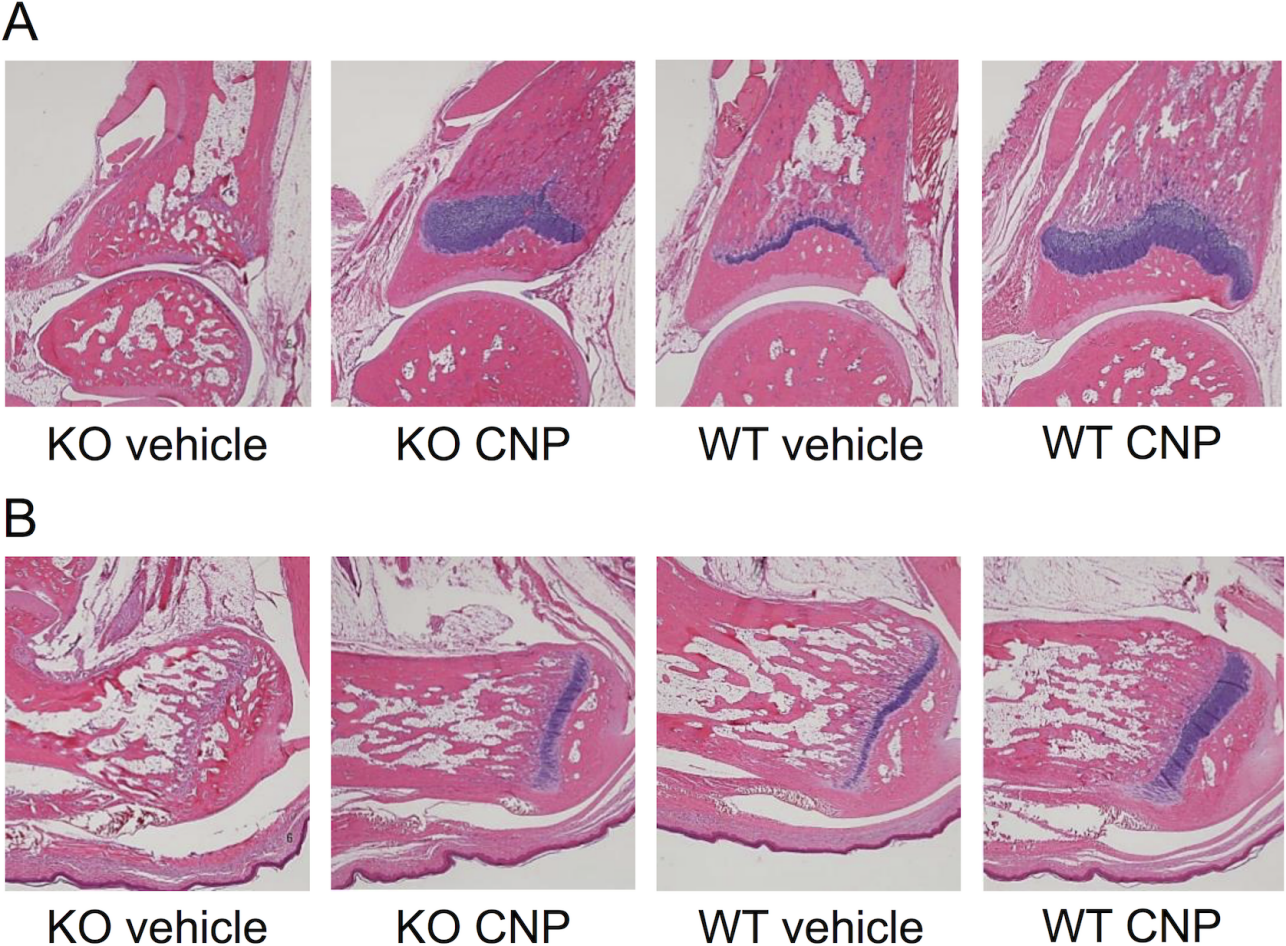
**Histological micrographs of the distal end of the tibia (A) and the calcaneus (B).** KO vehicle, CNP-KO rats that received vehicle; KO CNP, CNP-KO rats that received CNP-53 (0.5 mg/kg/day); WT vehicle, WT rats that received vehicle; and WT CNP, WT rats that received CNP-53 (0.5 mg/kg/day). HE and further alcian blue staining for growth plate were used.

Growth plate morphology was analyzed in the CNP-KO and WT rats that received high dose treatment of CNP-53, or vehicle. After the four-week experimental period, the growth plates were evaluated at the proximal and distal ends of the femur and tibia, and at the calcaneus. At the proximal and distal ends of femur, and at the proximal end of tibia, the growth plate thickness in the vehicle-treated CNP-KO rats was significantly narrower, at 64–72% of those in the vehicle-treated WT rats (Table 3). As shown in Fig 4 and Table 3, the growth plates at the distal end of tibia and the calcaneus were closed in all the CNP-KO rats that received vehicle, and CNP-53 treatment prevented the closure of growth plate in all the rats. The average thickness of growth plates at all the sites evaluated was significantly larger in the CNP-53 treated CNP-KO rats compared with those that received vehicle (Table 3). As seen in Table 3, CNP-53 treatment also significantly increased the growth plate thickness in the WT rats.

**Table 3:** Growth plate thickness at the proximal and distal ends of the femur and tibia, and at the calcaneus in the CNP-KO and WT rats.

Geno type	CNP-53 Dose (mg/kg/day)	n	Femur (μm)						Tibia (μm)						Calcaneus (μm)		
			proximal			distal			proximal			distal					
WT	0 (Vehicle)	5	110.4	±	3.0	283.0	±	14.9	309.2	±	7.5	117.9	±	7.5	120.1	±	6.3
	0.5	6	249.3	±	13.8**	655.6	±	61.6**	424.7	±	41.6*	835.9	±	130.3**	252.0	±	14.4**
CNP-KO	0 (Vehicle)	5	79.4	±	3.9**	203.1	±	6.5**	198.4	±	3.8**		0****	0**		
	0.5	6	190.9	±	26.7^##^	507.5	±	86.9^##^	373.1	±	59.3^##^	477.6	±	100.4^##^	213.5	±	29.7^##^

Each value represents the mean ± SE. The number “0” means closure of the growth plates in all the rats in that group.

*, *p* < 0.05 vs. WT rats received vehicle

**, *p* < 0.01 vs. WT rats that received vehicle

^##^, *p* < 0.01 vs. CNP-KO rats that received vehicle, based on the Student’s *t*-test.

**Fig 5:**
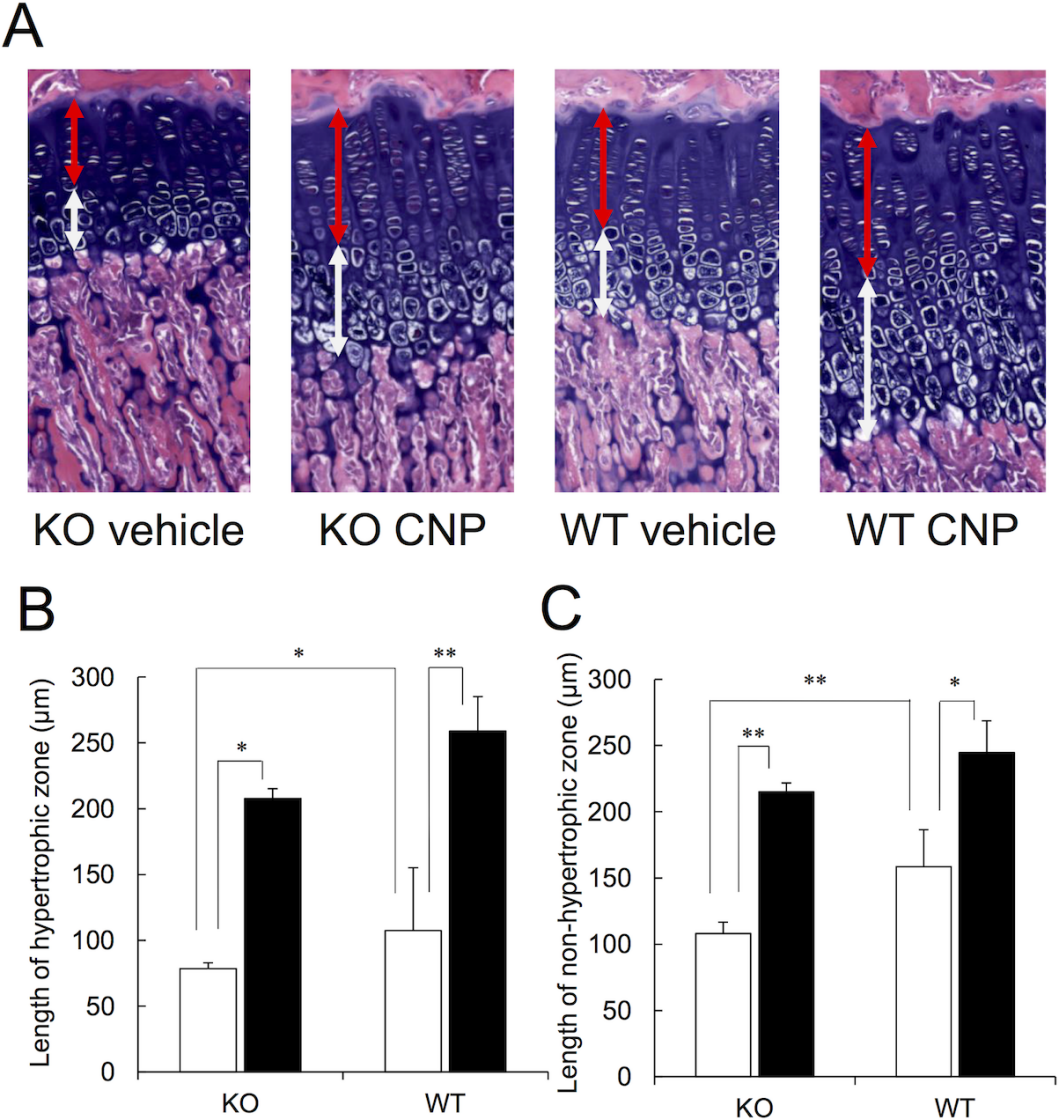
Effect of CNP-53 on the thickness of the hypertrophic and non-hypertrophic zones in the growth plate at the proximal end of the tibia. (A) Representative example of the proximal end of the tibia stained with alcian blue/HE. The white and red arrows indicate the hypertrophic and non-hypertrophic zones, respectively. (B), (C) The average lengths of the hypertrophic (B) and non-hypertrophic (C) zones. The values are the means + SE. Open bars, vehicle (n = 5 in both genotypes); closed bars, CNP-53 (0.5 mg/kg/day, n = 6 in both genotypes). KO, CNP-KO rats; WT, WT rats. *, *p* < 0.05, ** *p* < 0.01 based on the Student’s *t*-test.

The thickness of the hypertrophic and non-hypertrophic zones of the growth plates at the proximal end of tibia was also measured. A representative example of the growth plate at the proximal end of tibia is shown in Fig 5A, where the growth plates were clearly thickened by treatment with CNP-53 in both the CNP-KO and WT rats. As shown in Fig 5B and 5C, the thickness of both the hypertrophic and non-hypertrophic zones at the proximal tibia was narrower in the CNP-KO rats compared with WT rats, and these were significantly elongated by CNP-53 treatment. In the WT rats, CNP-53 treatment also significantly thickened both the hypertrophic and non-hypertrophic zones.

### Bone microstructure after CNP-53 treatment

**Fig 6:**
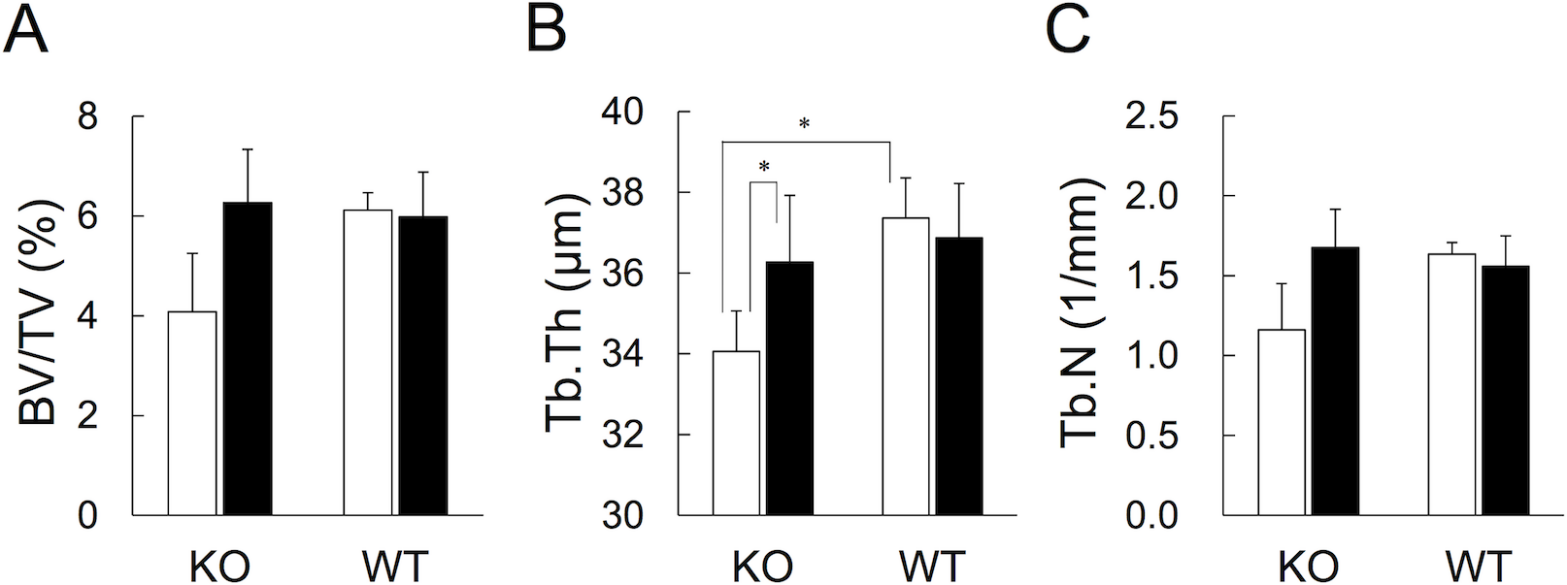
Effect of CNP-53 on bone microstructure at the distal end of the femur. (A) The bone volume density (BV/TV), (B) trabecular thickness (Tb.Th), and (C) trabecular number (Tb.N). The values are the means + SE. Open bars, vehicle (n = 5 in both genotypes); closed bars, CNP-53 (0.5 mg/kg/day, n = 6 in both genotypes). KO, CNP-KO rats; WT, WT rats. *, *p* < 0.05 vs. CNP-KO rats that received vehicle based on the Student’s *t*-test.

At the end of the administration period of CNP-53 or vehicle, the bone volume density (BV/TV), trabecular thickness (Tb.Th), and trabecular number (Tb.N), measured at the distal end of the femur, were lower in CNP-KO rats that received vehicle, compared with WT rats that received vehicle, and the significant difference was observed in Tb.Th (Fig 6). In addition, treatment with CNP-53 increased the average values for all the above mentioned parameters, but the significant increase was observed only in Tb.Th.

### Plasma bone turnover markers

**Fig 7:**
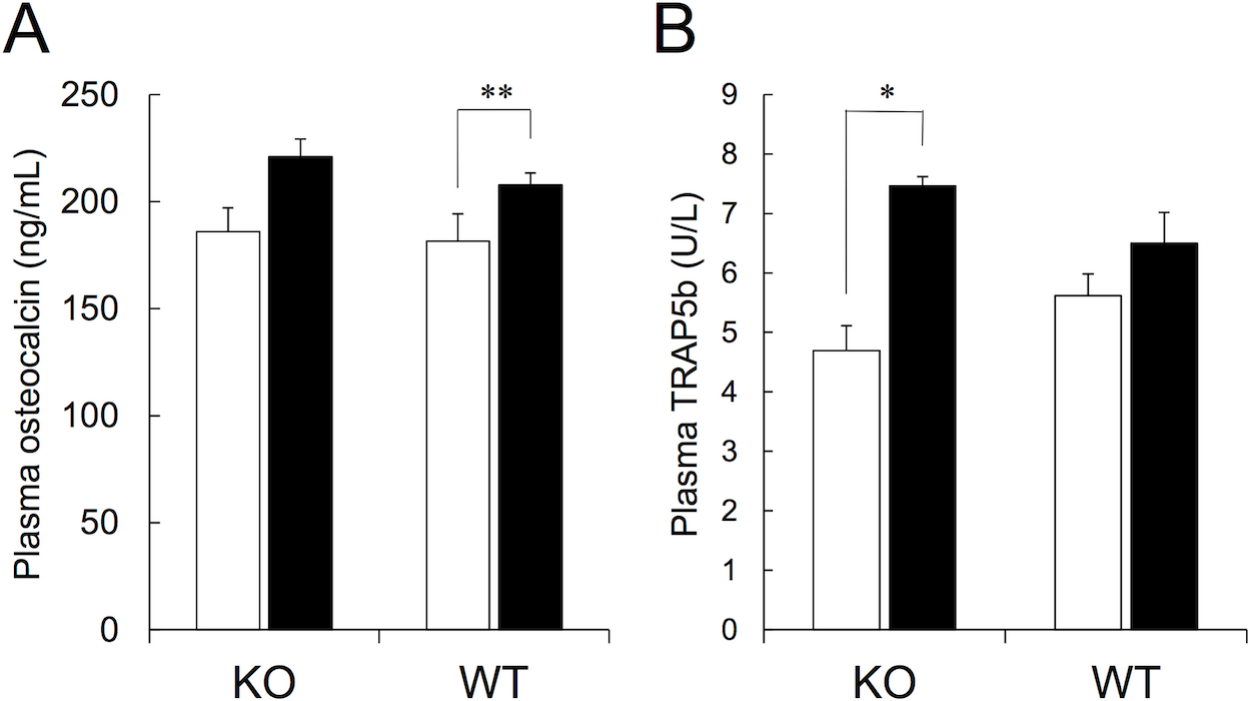
Effect of CNP-53 on bone turnover markers. (A) Plasma osteocalcin levels, and (B) plasma TRAP5b levels. The values are the means + SE. Open bars, vehicle (n = 5 in both genotypes); closed bars, CNP-53 (0.5 mg/kg/day, n = 6 in both genotypes). KO, CNP-KO rats; WT, WT rats. *, *p* < 0.05 vs. CNP-KO rats that received vehicle, and **, *p* < 0.01vs. WT rats that received vehicle based on the Student’s *t*-test.

We measured plasma levels of indicators for bone turnover in CNP-KO and WT rats that received vehicle or the high dose of CNP-53. Plasma osteocalcin and TRAP5b were evaluated as markers for bone formation and bone resorption, respectively. Both plasma osteocalcin and TRAP5b levels were similar in the CNP-KO and WT rats that received vehicle. The values for these parameters were higher in the CNP-53-treated groups of both the CNP-KO and WT rats, and statistically significant increases were detected for plasma osteocalcin in the WT rats and plasma TRAP5b in the CNP-KO rats (Fig 7).

### Expression of mRNA for natriuretic peptide receptors and ligands in CNP-KO rats

**Fig 8:**
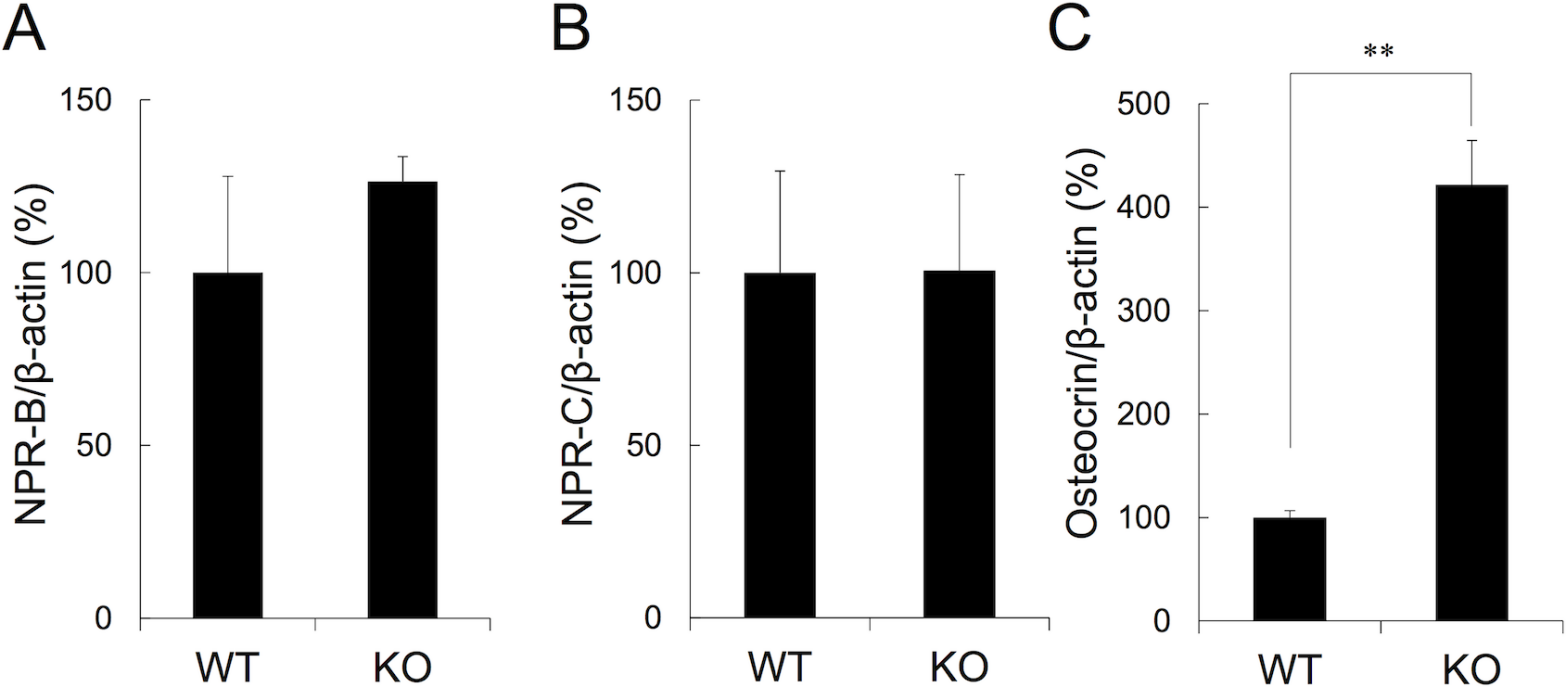
mRNA expressions for NP receptors and osteocrin in CNP-KO rats. (A) NPR-B mRNA, (B) NPR-C mRNA, and (C) osteocrin mRNA. The values are the means + SE (n = 3) and normalized using β-actin as an internal control. The average values in WT rats were regarded as 100%. **, *p* < 0.01 based on the Student’s *t*-test.

We investigated the effect of CNP deficiency on the expression of mRNA for the CNP receptors, NPR-B and NPR-C in the growth plates. Quantitative RT-PCR revealed that the expression of mRNAs for NPR-B and NPR-C was similar among the genotypes (Fig 8A and 8B). As for the natriuretic peptide ligands concerning CNP regulation, mRNA for osteocrin, an endogenous NPR-C ligand [19], was four times higher in the growth plate of CNP-KO rats than in WT rats (Fig 8C).

### CNP-53 has a negative influence on MAPK/ERK signaling pathway

**Fig 9:**
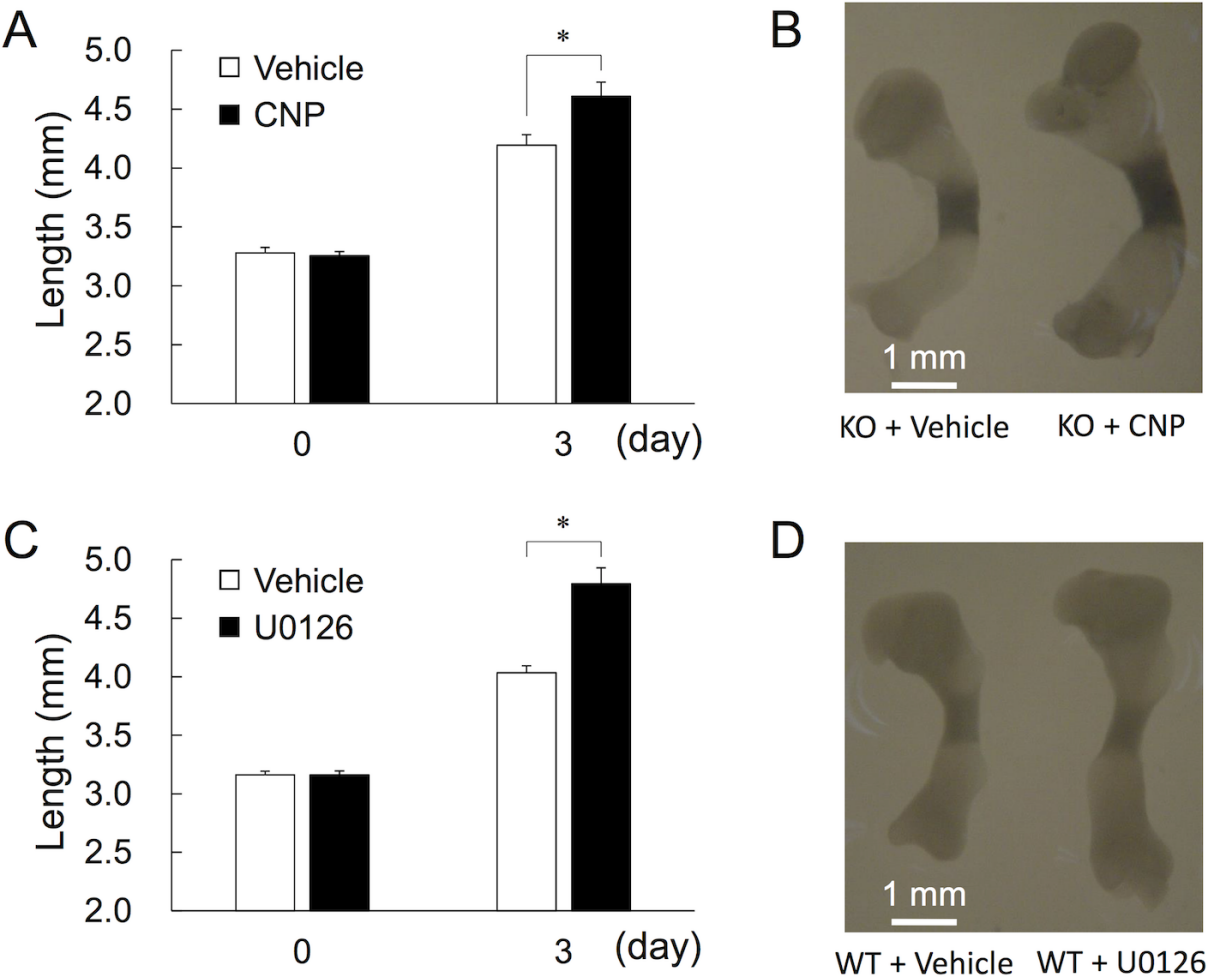
Effects of CNP-53 and U0126 on cultured tibiae from fetal rats. (A) Tibial lengths from fetal CNP-KO rat (E18.5) at the beginning (day 0) and at the end of culture period (day 3) with or without CNP. n = 6 in the both groups during the experimental period. *, *p* < 0.05 vs. vehicle (DMSO) based on Student’s *t*-test. (B) The representative picture of cultured tibiae from CNP-KO rats incubated with or without CNP. (C) Tibial lengths from fetal WT rat (E18.5) at the beginning (day 0) and at the end of culture period (day 3) with or without U0126. n = 6 in the both groups during the experimental period. *, *p* < 0.05 vs. vehicle (DMSO) based on Student’s *t*-test. (D) The representative picture of cultured tibiae from WT rats incubated with or without U0126.

Next, we attempted to identify which cellular signaling pathways are perturbed by CNP. To minimize the effect of endogenous CNP, we utilized tibial organ culture system from fetal CNP-KO rats at E18.5. As shown in Fig 9A and 9B, CNP significantly elongated cultured tibiae from fetal CNP KO rats. Since previous studies suggest that CNP inhibits MAPK/ERK signaling cascade [5], we next tried to test the functional correlation of CNP-53 with U0126, a known MEK1/2 inhibitor. Firstly, we confirmed that U0126 significantly stimulated the growth of cultured tibiae from fetal WT rats at the same gestational age, which was consistent with the previous study [22] (Fig 9C and 9D). Next, we performed microarray analyses using total RNA extracted from hypertrophic zones of tibial explants from fetal CNP-KO rats treated with or without CNP and those from WT rats treated with or without U0126. The results of microarray analyses are shown in S2–S5 Tables, which exhibit the lists of the top 50 up- or down-regulated genes by CNP in CNP-KO material and by U0126 in WT material. Then, using microarray data, we performed gene set enrichment analysis (GSEA) to examine whether U0126-responsible genes, i.e., genes differentially expressed upon U0126 treatment, positively correlated with CNP-53 target genes. The gene expression profile upon CNP-53 treatment was positively correlated with U0126-responsible genes, especially those down-regulated by U0126 (*p* < 0.001) (S1 Fig). These results suggest that CNP-53 causes similar transcriptome changes as U0126 on a genome-wide scale, and may target MAPK signaling cascades, especially MEK1/2.

## Discussion

In the current study, we evaluated the growth and skeletal phenotypes of CNP-KO rats and the therapeutic effect of CNP-53 in these CNP-deficient rats. We demonstrated that CNP-KO rats could be used as a reproducible animal model for skeletal dysplasia due to their impairment in endochondral bone growth, and the fact that they also exhibited early growth plate closure. This is the first report evaluating the skeletal growth effects of CNP-53 *in vivo*, the tissue form of CNP [16], by administering a sustained treatment for up to four weeks. The CNP-53 promoted a dose-dependent increase in growth in both the CNP-KO and WT rats, with the CNP-KO rats being more sensitive to the CNP-53 treatment. The CNP-53 also normalized the length of long bones and prevented growth plate closure of distal tibia and calcaneus in the CNP-KO rats. According to our gene set enrichment analysis, the inhibition of MAPK/ERK pathway might play an important part in the promoting effect of CNP on endochondral bone growth.

We and the others have suggested the therapeutic potential of CNP or its analog for the treatment of skeletal dysplasia such as achondroplasia [2–8, 17]. In the present study, we generated recombinant CNP-53, one of the endogenous forms of CNP, and evaluated its effect on growth of the animals, and growth plate histology in CNP-KO and WT rats. We selected CNP-53 because it is considered the dominant endogenous form of CNP in tissues including the bone [16] and has a higher NEP resistance compared to that of CNP-22 [17]. Previously, we utilized CNP-22 to stimulate skeletal growth in mice, but it required continuous intravenous infusion to promote skeletal growth, possibly due to its low metabolic stability *in vivo* [7].

As described above, CNP-53 stimulated skeletal growth in CNP-KO rats in a dose-dependent manner. At the low dose of 0.15 mg/kg/day, the increase in body length was significantly improved compared with the vehicle-treated group, and was similar to that in the WT rats that received vehicle (Fig 2, Table 2). This indicated that the low dose of CNP-53 induced normal growth rates in the CNP-KO rats. At the high dose of 0.5 mg/kg/day, the growth rates of CNP-KO rats exceeded those of WT rats, and the final body length was almost the same as that of WT rats that received vehicle. In other words, the high dose of CNP-53 was able to induce “catch-up growth” in the CNP-KO rats. The plasma CNP-53 concentration at the end of the four-week period of constant sc infusion of 0.5 mg/kg/day was approximately one ng/mL (around 170 pM) in both CNP-KO rats and WT rats. Even lower levels likely achieved by 0.15 mg/kg/day would be effective, as plasma CNP-22 levels ranging from 3–5 pM are associated with strong growth plate expansion induced by growth hormone stimulation in its deficient rats [24]. The normal growth rate and catch-up growth were achieved by the infusion of CNP-53 for only four weeks. A longer treatment study with CNP-53 may more precisely reveal the therapeutic ranges of plasma concentration required for skeletal growth.

In the current study, constant sc infusion of CNP-53 induced skeletal growth in a dose-dependent manner in both the CNP-KO and WT rats (Table 2, Fig 1 and Fig 2). We also confirmed that the effect of CNP-53 on rat growth was independent of sex (Table 1). Notably, the effects of CNP-53 were more prominent in the CNP-KO rats compared with those in the WT rats. This phenomenon might at least in part owe the fact that the mRNA for osteocrin, a natural NPR-C ligand [19], was increased in the growth plates of CNP KO rats compared to WT rats, whereas there were no differences in mRNA expressions for NPR-B and NPR-C between genotypes (Fig 8), even though the lines and the gender of CNP KO rats used in administration experiments and RT-PCR analyses were different (homozygous Δ 11 deleted female and homozygous Δ 774 deleted male, see “Materials and methods”); the increased osteocrin in CNP-KO rats might prevent the degradation of exogenous CNP-53 and contribute to the enhanced skeletal growth in these rats compared with WT rats [25]. Since the plasma CNP concentration was similar in CNP-KO and WT rats after four weeks of CNP-53 infusion, the increase of CNP-53 at growth plate through the local regulation by osteocrin may lead to the stronger effect on skeletal growth in CNP-KO rats. On the other hand, the CNP treatment has no effect on radial length in WT rats (Fig 3). The reason is not known, but the growth stimulating effect of CNP tended to be stronger in lower limbs and vertebrae than in upper limbs; the expression patterns of CNP receptors might be changed or the effect of CNP might be weakened by some unknown reasons. Further studies would be needed to elucidate this issue.

CNP-53 dose-dependently also increased the body weight gain in CNP-KO rats, but not in WT rats (Fig 2, Table 2). Previously, we crossed CNP-KO mice with liver specific CNP-Tg mice, which promotes improvement in both longitudinal growth and body weight [8]. The effect of CNP-53 on body weight gain is a secondary outcome to recovering skeletal growth, and together these results suggest that CNP-53 treatment may normalize the overall growth in CNP-KO rats.

One of our novel findings in CNP-KO rats was the early closure of the growth plate observed at some growth plates; this phenotype was observed at the proximal and distal ends of femurs at 33 weeks of age in our previous study [14]. In the current study, we found that the growth plates at the distal end of the tibia and the calcaneus were already closed in the CNP-KO rats at eight weeks of age (Table 3, Fig 4). The mechanism of early growth plate closure in the CNP-KO rats is still unclear, but the impairment in the endochondral bone growth may be involved. Agoston et al. reported that CNP stimulates endochondral bone growth and delays bone matrix formation and ossification via p38 MAPK signaling pathway [22]. They also reported that CNP reciprocally induces the expression of its clearance receptor, NPR-C, and Esapa et al. further showed the delayed bone matrix formation *in vivo* in mice with loss of function mutation in the NPR-C gene via p38 MAPK pathway [26]. In humans, acromesomelic dysplasia, type Maroteaux (AMDM) is caused by biallelic mutations in the gene encoding NPR-B, the bioactive receptor for CNP [9]. A review paper describes that the acromesomelic dysplasia is characterized by the abnormal/premature fusion of the growth plate in long bones, resulting in disproportionately shortened forearms, forelegs, hands, and feet [27], and the AMDM phenotype includes Madelung deformity, which is the asymmetric premature closure of the distal radial and ulnar growth plates. The clinical importance of the early growth plate closure should be considered and evaluated in the future in individuals with CNP mutations, NPR-B mutations, or other types of skeletal dysplasia with mutations in the genes relevant to the CNP/NPR-B signaling including NPR-C [26].

Furthermore, it is noteworthy that CNP-53 prevented growth plate closure (Table 3, Fig 4). As mentioned above, we have previously observed that the growth plates at the proximal and distal ends of the femur are closed in CNP-KO rats at 33 weeks of age [14]. If a longer-term treatment with CNP-53 or another CNP analog is able to delay the growth plate closure at any long bones including the femur, it will provide a longer growth period and may improve final height. The effect of CNP on the growth plate closure would be of clinical interest, since precocious puberty is a known cause of short stature following earlier puberty due to premature closure of growth plates in long bones [28]. As mentioned, the exact mechanisms of growth plate closure are still unclear in both rodents and humans, but estrogen is believed to play a role by advancing growth plate senescence [29]. Although there are some differences in growth plate physiology between rodents and humans, and there are difficulties in the direct application to humans of results from growth plate studies in rodents [29], CNP-KO rats might prove a valuable animal model for investigating growth plate physiology and the mechanism of closure.

The result of the bone microstructure analysis of the CNP-KO rats and its alteration after CNP-53 treatment are controversial. We think it noteworthy that the bone volume in the CNP-KO rats tended to be lower than the WT rats and recovered by the CNP supplementation. In our previous report, we showed that the bone mineral density of the whole femur of the CNP-KO rats was almost identical to that of the WT rats [14]. Apart from the averaged bone density in a whole long bone, the trabecular bone volume just beneath the growth plate would be decreased in the CNP-KO rats compared to the WT rats, and the supplementation of CNP might reverse it. On the other hand, in WT rats, the administration of CNP did not affect, or showed a slight tendency of the decrease in bone volume and increased the bone turnover markers, which was fundamentally coincided with the results of our previous study using a mouse model [30]; the limited effect in the present study might be due to the limitation of the experimental period or the dose of administration.

Recently, Hisado-Oliva et al. reported the loss of function mutations in the CNP gene in humans for the first time; in that report, they showed the heterozygous mutations cause short stature and brachydactyly [11]. We showed the growth pattern of heterozygous CNP-KO rats in our previous report [14], but it would be important to observe the effect of the CNP-treatment on the growth and growth plate morphology of the heterozygous CNP-KO rats: it would be good to investigate that there is no interference of this treatment when having half dosage values of endogenous CNP. Accordingly, we are now planning to study this issue using the heterozygous CNP-KO rats.

MAPK/ERK pathway plays important roles in the process of endochondral ossification. Activation of MAPK/ERK and Stat1 pathways through FGF receptor 3 (FGFR-3) have reported to inhibit proliferation and hypertrophic differentiation of chondrocytes and result in a decrease of the width of proliferative and hypertrophic zones [31]. Activation of Stat1 pathway is presumed to inhibit proliferation of chondrocytes, whereas, activation of MAPK/ERK pathway is considered to inhibit hypertrophic differentiation [32], and conversely, inactivation of ERK 1/2 in chondrocytes have reported to promote hypertrophic differentiation [33], that is, MAPK/ERK pathway is supposed to be a negative regulator of the hypertrophic differentiation of the chondrocytes. We previously reported that overexpression of CNP in chondrocytes counteracts dwarfism of an achondroplasia model mouse with activated FGFR-3 in the cartilage, and exhibited that inhibition of the MAPK/ERK pathway is one of the mechanism of an antagonistic effect of CNP to FGFR-3 signaling [5]. Therefore, it is meaningful to compare the gene expression patterns of the CNP-treated hypertrophic chondrocytes with those of U0126-treated ones, in order to investigate the mechanism of the stimulating effects of CNP on endochondral bone growth. In the current study, especially, we compared mRNA from tibial explants from CNP KO rats with that treated with CNP, to exaggerate exogenous CNP effects. Resultantly, our GSEA results showed that U0126-treated gene set was positively correlated with CNP-53–treated gene set, especially that concerns with down-regulated genes (S1 Fig). Taken the result of bone elongation by either CNP or U0126 supplementation in our organ culture experiment together (Fig 9), the inhibition of MAPK/ERK pathway may play an important role, at least in part, in the promoting effects of CNP on endochondral bone growth.

In conclusion, we confirmed that CNP-KO rats are applicable as reproducible animal models of growth disorders with early growth plate closure, and that CNP-53 significantly restored growth and prevented growth plate closure. CNP-53 treatment induced a normal growth rate at a lower dose, and catch-up growth at a high dose with four weeks of sc infusion. Longer treatment with CNP-53 would provide a more precise therapeutic concentration range to affect growth plate closure at various sites, and ultimately influence on the final height.

## Supporting information

S1 Table The plasma CNP concentrations at steady state in the CNP-KO and WT rats treated with CNP-53 at the dose of 0.5 mg/kg/day. (PDF)

S2 Table The list of the top 50 up-regulated genes by CNP in CNP KO hypertrophic zone. (PDF)

S3 Table The list of the top 50 down-regulated genes by CNP in CNP KO hypertrophic zone. (PDF)

S4 Table The list of the top 50 up-regulated genes by U0126 in WT hypertrophic zone. (PDF)

S5 Table The list of the top 50 down-regulated genes by U0126 in WT hypertrophic zone. (PDF)

S1 Fig GSEA of CNP-53 for gene sets related to U0126. Genes were ranked by fold change in a DNA micro array experiment (CNP-53 vs. vehicle). The top 100 up (A)/down (B) genes from U0126 treatment in WT rat tibiae were analyzed. NES, normalized enrichment score; FDR, false discovery rate.
(PDF)
