# Supplementary material for: Exogenous C-type natriuretic peptide restores normal growth and prevents early growth plate closure in its deficient rats
Source: PLoS One. 2018 Sep 20;13(9):e0204172. doi: 10.1371/journal.pone.0204172 (PMC6147488; doi:10.1371/journal.pone.0204172)
Supplement: S1 Table — (PDF) [file pone.0204172.s001.pdf]

S1 Table. The plasma CNP concentrations at steady state in each CNP-KO and WT rat treated with CNP-53 at the dose of 0.5 mg/kg/day.

genotype	ng/mL
CNP KO	1.14
CNP KO	2.44
CNP KO	0.39
CNP KO	0.50
WT	1.07
WT	2.44
WT	0.73
WT	1.92
WT	0.69
