# Supplementary material for: Exogenous C-type natriuretic peptide restores normal growth and prevents early growth plate closure in its deficient rats
Source: PLoS One. 2018 Sep 20;13(9):e0204172. doi: 10.1371/journal.pone.0204172 (PMC6147488; doi:10.1371/journal.pone.0204172)
Supplement: S2 Table — (PDF) [file pone.0204172.s002.pdf]

S2 Table. The list of the top 50 up-regulated genes by CNP in CNP-KO hypertrophic zone.

Gene Name	Gene Description	Fold Change (CNP/Vehicle)
Slmap	sarcolemma associated protein	55.0524
Clec5a	C-type lectin domain family 5, member A	51.5188
LOC100912790	nidogen-2-like	47.0335
LOC100913013	retinoic acid early-inducible protein 1-beta-like	44.4419
Atp2b3	ATPase, Ca++ transporting, plasma membrane 3	30.0791
Ptch2	patched homolog 2 (Drosophila)	29.7308
Olr1076	olfactory receptor 1076	28.9217
LOC498316	hypothetical LOC498316	27.6117
Clcn4	chloride channel, voltage-sensitive 4	23.3157
Olr1525	olfactory receptor 1525	22.3545
Klhdc7a	kelch domain containing 7A	21.9363
Tlr12	toll-like receptor 12	21.1878
Tex12	testis expressed 12	20.8229
Olr63	olfactory receptor 63	20.1128
Ttc23	tetratricopeptide repeat domain 23	17.7277
Lingo4	leucine rich repeat and Ig domain containing 4	17.3838
LOC688459	hypothetical protein LOC688459	17.2448
Cnga1	cyclic nucleotide gated channel alpha 1	17.0807
Tktl2	transketolase-like 2	17.0615
LOC498793	similar to inter-alpha-inhibitor H2 chain	16.3048
H2afy	H2A histone family, member Y	16.007
Camk2d	calcium/calmodulin-dependent protein kinase II delta	16.0019
Gpx2	glutathione peroxidase 2	15.2409
Scel	sciellin	14.6358
Krt9	keratin 9	14.559
Klhl34	kelch-like 34 (Drosophila)	14.4956
Olr1158	olfactory receptor 1158	14.4373
Kcnj9	potassium inwardly-rectifying channel, subfamily J, member 9	14.3359
Sox9	SRY-box containing gene 9	14.0372
Wnt8a	wingless-type MMTV integration site family, member 8A	13.9761
Mug1	murinoglobulin 1	13.9299
Cers6	ceramide synthase 6	13.7742
RGD1309036	hypothetical LOC292874	13.7293
LOC100912350	zinc finger protein 507-like	13.6059
Olr444	olfactory receptor 444	13.4206
RGD1305627	hypothetical LOC314467	12.3578
Heca	headcase homolog (Drosophila)	12.2449
Trim50	tripartite motif-containing 50	12.1699
LOC680934	ribosomal protein L26 pseudogene	12.1173
Stab2	stabilin 2	12.0797
Cplx4	complexin 4	11.8102
Bpifa2	BPI fold containing family A, member 2	11.4101
Dsg3	desmoglein 3	10.9764
Reep1	receptor accessory protein 1	10.9598
Olr1164	olfactory receptor 1164	10.8973
Adad1	adenosine deaminase domain containing 1 (testis specific)	10.7515
Wipf2	WAS/WASL interacting protein family, member 2	10.6381
LOC361016	similar to RIKEN cDNA 4933406L09	10.5896
Nr1d1	nuclear receptor subfamily 1, group D, member 1	10.5201
Olr162	olfactory receptor 162	10.5081
