# Supplementary material for: Exogenous C-type natriuretic peptide restores normal growth and prevents early growth plate closure in its deficient rats
Source: PLoS One. 2018 Sep 20;13(9):e0204172. doi: 10.1371/journal.pone.0204172 (PMC6147488; doi:10.1371/journal.pone.0204172)
Supplement: S3 Table — (PDF) [file pone.0204172.s003.pdf]

S3 Table. The list of the top 50 down-regulated genes by CNP in CNP-KO hypertrophic zone.

Gene Name	Gene Description	Fold Change (CNP/Vehicle)
Fgf3	fibroblast growth factor 3	-99.8154
Ush2a	Usher syndrome 2A (autosomal recessive, mild)	-94.8305
Dnahc3l	dynein, axonemal, heavy chain 3-like	-66.29
LOC100363993	testis expressed gene 16-like	-63.7398
Add2	adducin 2 (beta)	-60.0827
Tnk1	tyrosine kinase, non-receptor, 1	-56.4145
Olr508	olfactory receptor 508	-52.9973
Olr215	olfactory receptor 215	-52.1303
Hbe1	hemoglobin, epsilon 1	-51.573
Afm	afamin	-50.5025
Grip2	glutamate receptor interacting protein 2	-49.4709
Vom2r42	vomeronasal 2 receptor, 42	-48.4194
Olr1240	olfactory receptor 1240	-48.3128
Cd74	Cd74 molecule, major histocompatibility complex, class II invariant chain	-47.5851
Trim17	tripartite motif-containing 17	-47.2975
Olr429	olfactory receptor 429	-46.6822
Gdpd4	glycerophosphodiester phosphodiesterase domain containing 4	-46.2217
Boll	bol, boule-like (Drosophila)	-43.9428
RGD1561963	similar to Dedicator of cytokinesis protein 10 (Protein zizimin 3)	-42.9365
LOC685171	similar to protein disulfide isomerase-associated 6	-42.9077
Hcrtr2	hypocretin (orexin) receptor 2	-42.1028
Inpp4b	inositol polyphosphate-4-phosphatase, type II	-41.788
Prf1	perforin 1 (pore forming protein)	-40.6963
Mcmdc2	minichromosome maintenance domain containing 2	-40.6125
Eaf2	ELL associated factor 2	-39.8513
Actbl2	actin, beta-like 2	-38.5165
Dscam	Down syndrome cell adhesion molecule	-37.45
Olr1206	olfactory receptor 1206	-36.3764
Hist1h1t	histone cluster 1, H1t	-33.9972
Tat	tyrosine aminotransferase	-31.1473
Rgs6	regulator of G-protein signaling 6	-30.9299
LOC691277	similar to Robo-1	-29.6374
Psca	prostate stem cell antigen	-28.8897
LOC100911204	protein CASC5-like	-28.5299
Rhbdd1	rhomboid domain containing 1	-28.2516
Aox3	aldehyde oxidase 3	-27.6002
Olr1306	olfactory receptor 1306	-27.4578
Lyzl1	lysozyme-like 1	-27.2065
Olr1320	olfactory receptor 1320	-26.749
LOC100912319	uncharacterized LOC100912319	-26.6903
N5	DNA binding protein N5	-26.6343
Olr856	olfactory receptor 856	-26.568
Map4	microtubule-associated protein 4	-26.5155
Fmo5	flavin containing monooxygenase 5	-25.6995
Ccl19	chemokine (C-C motif) ligand 19	-25.145
Amy2	amylase 2, pancreatic	-24.8396
Soga2	SOGA family member 2	-24.3534
Olr1679	olfactory receptor 1679	-23.6693
Dbc1	deleted in bladder cancer 1	-23.612
Sim1	single-minded homolog 1 (Drosophila)	-23.4706
