# Supplementary material for: Exogenous C-type natriuretic peptide restores normal growth and prevents early growth plate closure in its deficient rats
Source: PLoS One. 2018 Sep 20;13(9):e0204172. doi: 10.1371/journal.pone.0204172 (PMC6147488; doi:10.1371/journal.pone.0204172)
Supplement: S4 Table — (PDF) [file pone.0204172.s004.pdf]

S4 Table. The list of the top 50 up-regulated genes by U0126 in WT hypertrophic zone.

Gene Name	Gene Description	Fold Change (U0126/Vehicle)
Timm8a2	translocase of inner mitochondrial membrane 8 homolog a2 (yeast)	15.0807
LOC301839	similar to Spindlin-like protein 2 (SPIN-2)	11.3205
RGD1563425	similar to FLJ35784 protein	10.0645
Clgn	calmegin	9.6866
LOC689986	hypothetical protein LOC689986	9.6028
Atp1b4	ATPase, (Na+)/K+ transporting, beta 4 polypeptide	9.4922
LOC686050	hypothetical protein LOC686050	9.4072
Krt13	keratin 13	9.2517
Arhgap36	Rho GTPase activating protein 36	7.6669
Lrrc39	leucine rich repeat containing 39	7.3427
RGD1561667	similar to putative protein kinase	6.9648
Slc30a10	solute carrier family 30, member 10	6.517
Nmu	neuromedin U	6.1008
Fa2h	fatty acid 2-hydroxylase	6.0602
LOC100911353	uncharacterized LOC100911353	6.0514
Btbd10	BTB (POZ) domain containing 10	5.9229
LOC367515	similar to RIKEN cDNA 1700081O22	5.8867
LOC100360872	protease 1-like	5.7604
Slc6a1	solute carrier family 6 (neurotransmitter transporter, GABA), member 1	5.7581
Slc38a11	solute carrier family 38, member 11	5.7025
Acsm5	acyl-CoA synthetase medium-chain family member 5	5.5188
Trpm1	transient receptor potential cation channel, subfamily M, member 1	5.5045
Gpx2	glutathione peroxidase 2	5.4981
LOC685321	similar to protease, serine, 28	5.494
Tmem116	transmembrane protein 116	5.4621
Slc7a11	solute carrier family 7 (anionic amino acid transporter light chain, xc-system), member 11	5.3952
Cma1	chymase 1, mast cell	5.0908
Nfasc	neurofascin	4.918
Il22	interleukin 22	4.8325
Ccdc164	coiled-coil domain containing 164	4.5013
Chrdl2	chordin-like 2	4.3051
LOC681382	hypothetical protein LOC681382	4.1905
Tapt1	transmembrane anterior posterior transformation 1	4.1198
Gja5	gap junction protein, alpha 5	4.1172
Capn13	calpain 13	3.9152
Fgf21	fibroblast growth factor 21	3.8628
Hfe2	hemochromatosis type 2 (juvenile)	3.8461
Il17b	interleukin 17B	3.8028
LOC498316	hypothetical LOC498316	3.7267
Ckmt2	creatine kinase, mitochondrial 2, sarcomeric	3.5494
LOC689207	similar to proteoglycan 4	3.5187
Cacng6	calcium channel, voltage-dependent, gamma subunit 6	3.5159
Maob	monoamine oxidase B	3.4642
Mybph	myosin binding protein H	3.4537
Myh3	myosin, heavy chain 3, skeletal muscle, embryonic	3.3899
Card9	caspase recruitment domain family, member 9	3.3655
Cyp1a1	cytochrome P450, family 1, subfamily a, polypeptide 1	3.359
Park7	parkinson protein 7	3.3157
Actn2	actinin alpha 2	3.2645
Rhbdd1	rhomboid domain containing 1	3.2012
