# Supplementary material for: Exogenous C-type natriuretic peptide restores normal growth and prevents early growth plate closure in its deficient rats
Source: PLoS One. 2018 Sep 20;13(9):e0204172. doi: 10.1371/journal.pone.0204172 (PMC6147488; doi:10.1371/journal.pone.0204172)
Supplement: S5 Table — (PDF) [file pone.0204172.s005.pdf]

S5 Table. The list of the top 50 down-regulated genes by U0126 in WT hypertrophic zone.

Gene Name	Gene Description	Fold Change (U0126/Vehicle)
Cd207	CD207 molecule, langerin	-21.1442
Lrg1	leucine-rich alpha-2-glycoprotein 1	-16.5186
Rasl10a	RAS-like, family 10, member A	-16.2818
Flt1	FMS-related tyrosine kinase 1	-15.6747
Abca4	ATP-binding cassette, subfamily A (ABC1), member 4	-14.1465
LOC684480	similar to butyrophilin 3	-13.8896
Gja4	gap junction protein, alpha 4	-13.3883
LOC502859	similar to T-cell activation kelch repeat protein	-13.247
Tmem194b	transmembrane protein 194B	-13.2064
Sez6	seizure related 6 homolog (mouse)	-12.8089
Gimap4	GTPase, IMAP family member 4	-12.7529
Pou4f2	POU class 4 homeobox 2	-12.5876
Sox7	SRY (sex determining region Y)-box 7	-12.5259
Tmem154	transmembrane protein 154	-12.2351
Ccl6	chemokine (C-C motif) ligand 6	-11.9151
Ccl19	chemokine (C-C motif) ligand 19	-11.2978
LOC100360457	formin 2-like	-10.2564
Dscr6	Down syndrome critical region homolog 6 (human)	-9.7168
Upp1	uridine phosphorylase 1	-9.1925
Rnase10	ribonuclease, RNase A family, 10 (non-active)	-9.0035
LOC685067	similar to guanylate binding protein family, member 6	-8.7144
Kcne3	potassium voltage-gated channel, Isk-related family, member 3	-8.486
Glod5	glyoxalase domain containing 5	-8.4535
Hoxa1	homeo box A1	-8.3422
Rap2ip	Rap2 interacting protein	-8.2383
Ephb1	Eph receptor B1	-7.8313
Siglec5	sialic acid binding Ig-like lectin 5	-7.6651
Ifi204	interferon activated gene 204	-7.6384
Il1a	interleukin 1 alpha	-7.6198
Myoc	myocilin	-7.581
Foxs1	forkhead box S1	-7.5799
Gpr4	G protein-coupled receptor 4	-7.4833
Nrarp	Notch-regulated ankyrin repeat protein	-7.468
Fam70a	family with sequence similarity 70, member A	-7.445
RGD1565161	similar to dendritic cell-derived immunoglobulin(Ig)-like receptor 1, DIgR1 - mouse	-7.3665
Ccl20	chemokine (C-C motif) ligand 20	-7.2212
Ch25h	cholesterol 25-hydroxylase	-7.0495
Olr991	olfactory receptor 991	-6.9504
Gna14	guanine nucleotide binding protein, alpha 14	-6.9016
C7	complement component 7	-6.8015
Dll4	delta-like 4 (Drosophila)	-6.7399
Tmeff2	transmembrane protein with EGF-like and two follistatin-like domains 2	-6.7033
Trpc6	transient receptor potential cation channel, subfamily C, member 6	-6.6688
RGD1565505	similar to Zinc finger protein GLIS3 (GLI-similar 3)	-6.6137
RGD1564865	similar to 20-alpha-hydroxysteroid dehydrogenase	-6.6117
Lrrc4c	leucine rich repeat containing 4C	-6.5832
Tnf	tumor necrosis factor	-6.5207
Inmt	indolethylamine N-methyltransferase	-6.4683
Ms4a6a	membrane-spanning 4-domains, subfamily A, member 6A	-6.4476
Npw	neuropeptide W	-6.4119
