# Supplementary material for: Exogenous C-type natriuretic peptide restores normal growth and prevents early growth plate closure in its deficient rats
Source: PLoS One. 2018 Sep 20;13(9):e0204172. doi: 10.1371/journal.pone.0204172 (PMC6147488; doi:10.1371/journal.pone.0204172)
Supplement: S1 Fig — Genes were ranked by fold change in a DNA micro array experiment (CNP-53 vs. vehicle). The top 100 up (A)/down (B) genes from U0126 treatment in WT rat tibiae were analyzed. NES, normalized enrichment score; FDR, false discovery rate. (PDF) [file pone.0204172.s006.pdf]

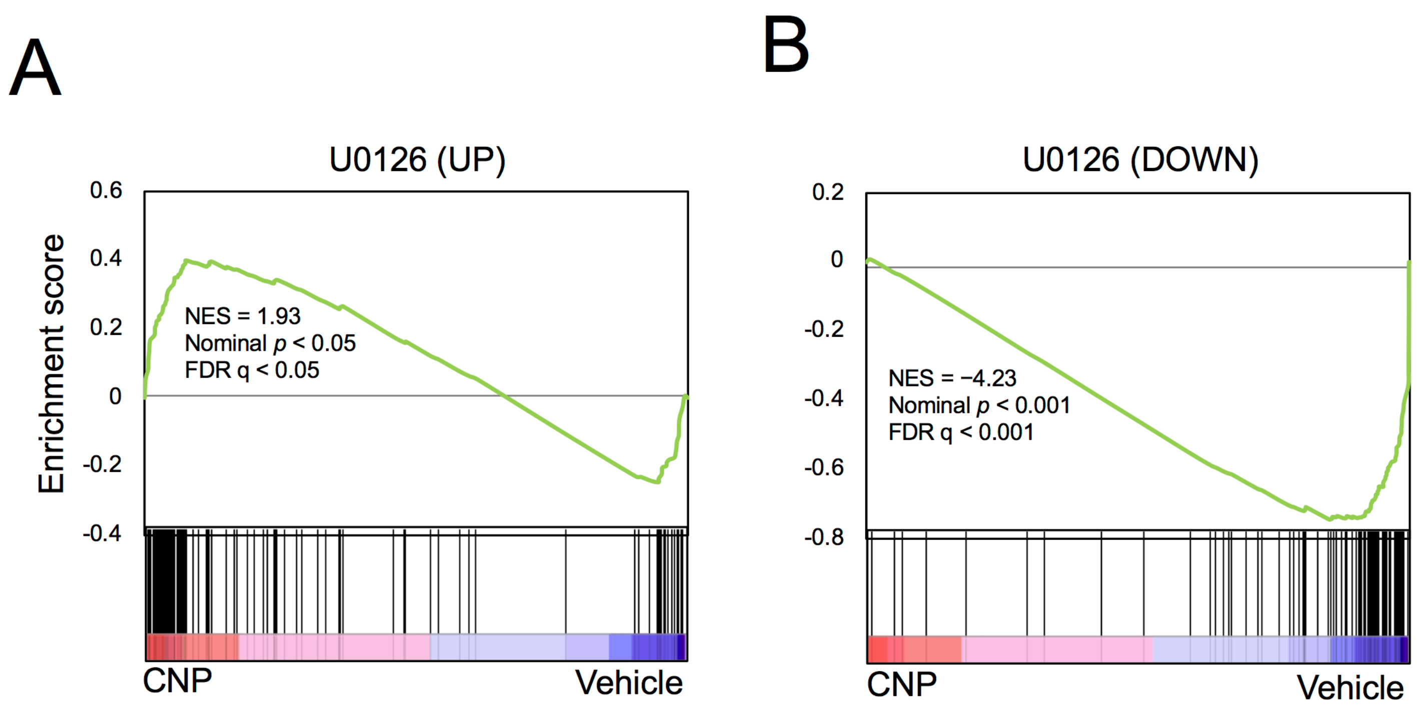

S1 Fig. GSEA of CNP-53 for gene sets related to U0126. Genes were ranked by fold change in a DNA micro array experiment (CNP-53 vs. vehicle). The top 100 up (A)/down (B) genes from U0126 treatment in WT rat tibiae were analyzed. NES, normalized enrichment score; FDR, false discovery rate.
